# Risk of Sequelae Following COVID-19 Infection: A Nationwide Study Focusing on Risk Factors and Long-Term Impacts

**DOI:** 10.3390/jcm14227950

**Published:** 2025-11-10

**Authors:** Simon Kjeldsen, Jan Nielsen, Bente Mertz Nørgård, Ken Lund, Pedro Póvoa, Torben Knudsen, Jens Kjeldsen

**Affiliations:** 1 Department of Pediatrics and Adolescent Medicine, Odense University Hospital, 5000 Odense, Denmark; 2 Center for Clinical Epidemiology, Odense University Hospital, 5000 Odense, Denmark; jan.nielsen2@rsyd.dk (J.N.); bente.noergaard@rsyd.dk (B.M.N.); pedrorpovoa@gmail.com (P.P.); 3 Department of Clinical Research, University of Southern Denmark, 5230 Odense, Denmark; jens.kjeldsen@rsyd.dk; 4 Intensive Care Unit 4, Department of Intensive Care, Hospital de São Francisco Xavier, Unidade Local de Saúde Lisboa Ocidental (ULSLO), Estrada do Forte do Alto do Duque, 1449-005 Lisbon, Portugal; 5 NOVA Medical School, Comprehensive Health Research Center (CHRC), New University of Lisbon, Campo dos Mártires da Pátria, 1099-085 Lisbon, Portugal; 6 Department of Medicine, Hospital of Southwest Jutland, 6700 Esbjerg, Denmark; torben.knudsen@rsyd.dk; 7 Department of Regional Health Science, University of Southern Denmark, 5230 Odense, Denmark; 8 Department of Medical Gastrointestinal Diseases, Odense University Hospital, 5000 Odense, Denmark

**Keywords:** COVID-19, sequelae, comorbidity, CCI

## Abstract

**Background/Objective**: The SARS-CoV-2 pandemic, emerging in late 2019, led to a global health crisis, with many patients developing prolonged symptoms after infection known as sequelae of COVID-19. This condition is theorized to be driven by systemic inflammation and immune dysregulation and presents with diverse symptoms from cardiovascular, pulmonary, and neurological systems. This study investigates the prevalence, risk factors, and long-term impacts of sequelae of COVID-19. **Method**: Using Denmark’s healthcare databases, this population-based cohort study included 1,034,093 individuals over 40 years who tested positive for COVID-19 between 1 March 2020 and 28 February 2022. Participants were divided into two age groups: 40–59 years and 60 years or older. Part A examined the risk of sequelae of COVID-19 diagnoses (ICD-10 code B94.8A) based on the Charlson Comorbidity Index (CCI). Part B assessed two-year outcomes for patients diagnosed with sequelae of COVID-19. **Results**: Results showed a 0.55% prevalence of sequelae of COVID-19 in both age groups. Higher CCI scores correlated with an increased risk of sequelae of COVID-19. During the two-year follow-up, patients with sequelae of COVID-19 faced significantly elevated risks of thromboembolic events, chronic lung diseases, and infections. Adjusted hazard ratios were notably high: 14.50 (7.54–27.86) and 12.50 (6.95–22.49) for thromboembolic events in adults and older adults, respectively; 33.81 (13.30–85.96) and 9.83 (6.09–15.87) for chronic lung disease; and 8.40 (4.49–15.70) and 15.44 (10.47–22.78) for infections. **Conclusions**: While the overall prevalence of sequelae of COVID-19 was low among individuals over 40, those with higher comorbidity burdens were at greater risk of severe sequelae and subsequent health complications. These findings underscore the need for clinical monitoring, especially for patients with pre-existing comorbidities, to mitigate long-term health risks associated with COVID-19 sequelae.

## 1. Introduction

On 31 December 2019, the first cases of SARS-CoV-2, later known as COVID-19, were reported from Wuhan, China [1]. The initial reports described a disease of varying severity, from asymptomatic cases to severe viral pneumonia requiring treatment in intensive care units [2,3]. The following pandemic led to a worldwide lockdown, and as of now, over 777 million cases have been confirmed and over 7 million deaths due to COVID-19 [4].

The first peer-reviewed articles describing long-lasting symptoms after COVID-19 were published in the summer of 2020 [5,6]. In 2021, the WHO defined sequelae of COVID-19 as: ‘usually 3 months from the onset of COVID-19 with symptoms that last for at least 2 months and cannot be explained by an alternative diagnosis’ [7]. In the following years, there has been an increasing focus on sequelae of COVID-19, with studies showing a wide range of cardiovascular, pulmonary, gastrointestinal, muscular, neurological, and psychological symptoms [8,9,10,11,12,13,14]. One study found that up to 60% of patients referred with sequelae of COVID-19 complain of dyspnoea [9] and while pulmonary fibrosis can explain part of the patients with dyspnoea following a COVID-19 infection [15], other studies have theorized that the aforementioned autoimmune dysfunction, as well as neuromuscular involvement, can likewise be part of the explanation [16]. Patients with sequelae of COVID-19 have been shown to exhibit ongoing systemic inflammation and immune dysregulation of T cells. An imbalance between the cellular and humoral immune response has been theorized as an underlying cause [17,18].

Furthermore, the link between thromboembolic events and acute COVID-19 infection is well established [19], where thrombotic endothelialitis, endothelial inflammation, and hyperactivated platelets have been theorized as underlying causes [20]. Finally, it has been documented that patients with sequelae of COVID-19 have a dysregulated thromboembolic system up to 6 months after infection, indicating a prolonged risk for thromboembolic complications [21].

Studies of patients following COVID-19 infection have demonstrated both short-term risks, with increased mortality immediately after infection [22,23] and long-term risks, with increased risk of morbidity and mortality, that persist up to two years post-infection [24]. Another study with long-term follow-up similarly showed increased risk of mortality with a three-year follow-up [25] and, furthermore, showed an increased risk of sequelae of COVID-19 following hospitalization compared to patients who were not hospitalized. While comprehensive, this study did not examine other risk factors of developing sequelae of COVID-19. Overall, a notable gap remains in long-term research assessing the risk of post-COVID sequelae years after the initial infection. While one study identified pre-existing comorbidities as a risk factor for developing sequelae, follow-up was limited to six months [26]. Our study seeks to deepen the understanding of the long-term risks associated with developing sequelae of COVID-19, utilizing nationwide registries and a two-year follow-up period.

The study was divided into two parts: In part A, we examined whether an increasing score of the Charlson comorbidity index (CCI) correlated with an increased risk of being diagnosed with sequelae of COVID-19. In part B, we examined the prognostic consequences for patients in Denmark who had a diagnosis of sequelae of COVID-19, and calculated their risk of infections, thromboembolic events, and chronic lung disease in a two-year follow-up after the diagnosis of sequelae of COVID-19.

## 2. Materials and Methods

### 2.1. Setting and Data

Denmark has a universal healthcare system with free access for all its approximately 5.9 million inhabitants, 90% of whom are of Caucasian ethnicity. All Danish citizens and permanent residents have a unique civil registration number, which enables cross-linkage of individual patients’ data between national databases. This homogeneous and standardized healthcare system, combined with mandatory registration by physicians in the national databases of Denmark, enabled us to use a nationwide study design [27] when setting up our cohort.

The data for the study were gathered through four nationwide databases. Laboratory analysis, registration, and release of the national SARS-CoV-2 surveillance data were conducted by the Danish Departments of Clinical Microbiology and Statens Serum Institut. From this database, data on individual patients’ first-time positive COVID-19 PCR test results were collected. Data on COVID-19 vaccination status were retrieved from the Danish vaccination register, which includes information regarding vaccination dates for the first, second, and third vaccinations for each individual patient, as well as brand of vaccine used [28]. The Danish National Patient Register (DNPR) was additionally employed; it contains longitudinal records of hospital admissions/discharges from 1977 onward and outpatient visits from 1995 onward. Among the data registered within DNPR are the underlying diagnoses for each patient contact as well as the date of admission and of discharge from the hospital. The diagnoses are registered with the Danish version of the International Classification of Diseases (ICD), with the 8th version being used up to 1994, whilst version 10 has been used since [29]. The Danish Civil Registration System was established in 1968, and holds the unique civil registration number granted at birth or upon immigration into the country, and includes data regarding sex, date of birth, death, and immigration status [30]. Each of these databases was cross-linked on an individual level using the unique civil registration number.

### 2.2. Study Population and Design

This population-based cohort study included all Danish residents over the age of 40 who had a positive COVID-19 PCR test between 1 March 2020 and 28 February 2022. Participants were grouped by age into two categories: 40–59 years and over 60 years. The follow-up period was two years from the date of their first positive COVID-19 PCR test. We only included patients 40 years and older in our study, as the focus of this study was the association between comorbidity and developing sequelae of COVID-19.

### 2.3. Exposure, References, and Outcomes

In part A, the exposure of interest was the CCI score as a metric for comorbidity. For the CCI, the diseases were identified using ICD-10 codes (in- and outpatients), and a 10-year lookback period from the index was applied [31,32]. Patients in each of the two age groups, 40–59 years and 60 years or older, were divided into the following exposed cohorts according to the severity of comorbidity: CCI 1–2 or CCI > 3. Patients with a CCI of 0 served as the reference group. The outcome was a diagnosis of sequelae of COVID-19 during a two-year follow-up after a positive COVID-PCR test. The outcome data were retrieved from DNPR based on either a primary or secondary diagnosis of ICD10: B948A (sequelae of COVID-19). The date of the first registration was used in case of multiple registrations. In Denmark, during the period our data were gathered, the diagnosis of sequelae of COVID-19 was only used by outpatient clinics in a hospital setting. Patients with severe symptoms were referred from their general practitioner either to the outpatient clinic of the relevant specialty if their symptoms were primarily from a singular organ system or a specialized multidisciplinary outpatient clinic, if multiple organ systems where involved. The diagnosis B948A Sequelae of COVID-19 was given to patients who exhibited neurological, cardiopulmonary and musculoskeletal symptoms as described by the National Institute for Health and Care Excellence (NICE). Furthermore, these symptoms had to have persisted or emerged at least 12 weeks after the initial COVID infection and not been explained by another underlying disease [33].

In part B, the exposed group included patients diagnosed with sequelae of COVID-19, while the reference group comprised patients who tested positive for COVID-19 by PCR but had no diagnosis of sequelae of COVID-19. As in part A, patients were divided into two age groups of 40–59 and 60 or above. The outcomes were diagnoses of thromboembolic disease, chronic respiratory disease, or infectious disease in a two-year follow-up after their first positive PCR test. The ICD codes used for each category are presented in Appendix A. If a patient had multiple ICD codes in the same category, only the first ICD code was used.

### 2.4. Confounders

Confounders were selected a priori and included age at the first PCR test as a continuous variable, sex, and the number of vaccinations prior to the first PCR test, categorized as one or two. The calendar time of the first PCR test was included as a confounder and divided into 6-month periods to reflect different variants of COVID-19. Additionally, any hospitalization due to COVID-19 was considered a confounder.

### 2.5. Statistical Analysis

In Part A, a descriptive table presenting frequencies and percentages for the main baseline variables was constructed for each age group of our population. The risk of having a diagnosis of sequelae of COVID-19 according to CCI was examined separately within each age group. A time-to-event approach was chosen, and patients were followed for up to two years from their first positive COVID-19 PCR test, until a diagnosis of sequelae of COVID-19 occurred, or a censoring date in case of emigration, death, or end of follow-up. From this, graphs displaying the cumulative incidence percentage of sequelae according to CCI score were constructed, based on the Kaplan–Meier estimate. A multivariable Cox proportional hazard regression was used to estimate crude and adjusted hazard ratios (HR) for the risk of a sequelae diagnosis, for CCI group 1–2 and CCI group 3+, with CCI of 0 as a reference. HRs were adjusted for the confounders sex, age, number of vaccinations, hospitalization, and calendar time. Since hospitalization can occur at any time after a positive test, it was constructed as a time-varying variable in the Illness-Death model [34]. Patients were considered “not hospitalized” before their hospitalization date and “hospitalized” from the time of admission onward.

Furthermore, as a supplementary analysis, we analyzed the specific impact of selected CCI disease categories. For each category, we compared patients with the specific disease category to patients without the specific category in a univariable model. Then, in a multivariable model, we included all chosen categories, as well as confounders, so HRs for the different CCI categories were mutually adjusted. Patients who had more than one CCI disease category were included in the analysis for each relevant category they belonged to.

In Part B, when analyzing the prognostic impact of a diagnosis of sequelae of COVID-19, we also used a multivariable Cox model, with sequelae of COVID-19 constructed as a time-varying exposure (yes/no). Hence, the time of a patient before being diagnosed with sequelae of COVID-19 contributed to the unexposed group, and the time after the diagnosis contributed to the exposed group. HRs were adjusted for the confounders mentioned above, as well as CCI. Hospitalization was again considered as a time-varying confounder. A follow-up period of 2 years after the initial positive PCR test was used in Part B. Stata 19 was used for all the statistical analyses [35].

## 3. Results

This study included 1,034,093 PCR-confirmed COVID-19 patients: 702,075 adults aged 40–59 years (67.9%) and 332,018 (32.1%) older adults, aged 60 years or older. Among adults, 52.9% (371,718) were female. Among older adults, 51.8% (171,857) were female.

In the adult group, 607,847 patients (86.6%) had a CCI score of zero, corresponding to no registered comorbidity, 83,642 (11.9%) had a CCI score of 1–2, and 10,586 (1.5%) had a CCI score of 3 or higher. In the older adult group, 202,782 patients (61.1%) had a CCI score of 0, 95,541 (28.8%) had a CCI score of 1–2, and 33,695 (10.1%) had a CCI score of 3 or higher. The most common comorbidity in both age groups was chronic pulmonary disease, affecting 23,117 patients (3.3%) of the adult group and 24,661 (7.4%) in the older adult group. The characteristics of each age group are shown in Table 1.

### 3.1. Sequelae of COVID-19 According to CCI

**Figure 1:**
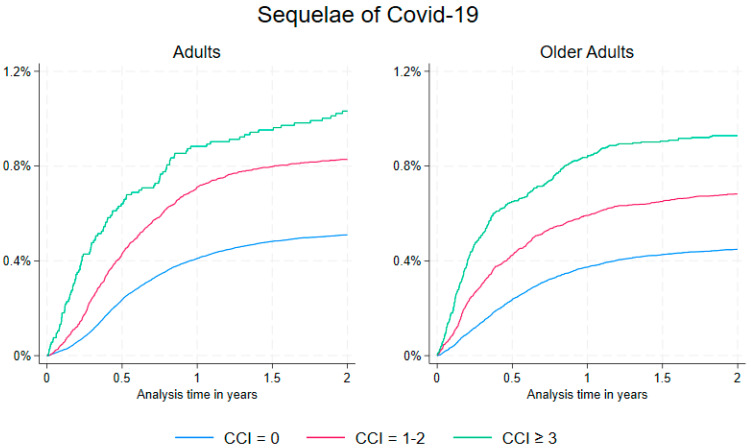
Cumulative incidence percentage of sequelae according to CCI score by adults (40–59 years, **left plot**) and older adults (≥60 years, **right plot**) in Danish PCR-positive COVID-19 patients with 2 years of follow-up.

The prevalence of sequelae of COVID-19 was 0.55% for both adults and older adults. When stratifying data by CCI category, the prevalence was 1% or lower across all three categories. Comparing patients with a CCI of 1–2 to the control patients with a CCI of 0, the adjusted risk estimate of being diagnosed with sequelae of COVID-19 was increased to 1.51 (95% CI: 1.39–1.64) for adults and 1.54 (95% CI: 1.39–1.71) for older adults. For patients with a CCI of 3 or higher, relative to those with a CCI of 0, the adjusted risk estimate was further elevated, 1.85 (95% CI: 1.52–2.24) in adults and 2.02 (95% CI: 1.76–2.32) in older adults. (See Table 2). The cumulative incidence of being diagnosed with sequelae of COVID-19 is visualized in Figure 1 for both age groups.

Analyzing the specific underlying diseases that constitute CCI scoring, we found that patients with chronic pulmonary disease and diabetes with chronic complications had the highest prevalence of being diagnosed with sequelae of COVID-19 in adults at 1.1%. In older adults, we found that patients with diabetes with chronic complications had the highest prevalence of sequelae of COVID-19 at 1.2%. When calculating the risk of being diagnosed with sequelae of COVID-19 for each individual underlying disease in the adult age group, only patients with chronic pulmonary disease and patients with rheumatologic disease had an isolated increased risk with HRs of 1.35 (95% CI: 1.19–1.53) and 1.25 (95% CI: 1.02–1.53), respectively. Similarly, in the older adults age group, only patients with chronic pulmonary disease and rheumatologic disease had an isolated increased risk of being diagnosed with sequelae of COVID-19 with HRs of 1.26 (95% CI: 1.10–1.44) and 1.40 (95% CI: 1.16–1.70), respectively. Conversely, dementia in older adults was associated with a decreased risk of being diagnosed with sequelae of COVID-19 with an HR of 0.36 (95% CI: 0.22–0.58). HRs for each corresponding CCI subgroup are presented in Supplement Table S1.

### 3.2. Diseases Following Being Diagnosed with Sequelae of COVID-19

Over a two-year period after a positive PCR test, we examined whether patients diagnosed with sequelae of COVID-19 had a higher risk of being admitted to the hospital with the following disease categories: thromboembolic event, respiratory disease, or infectious diseases, compared to patients with COVID-19 who did not suffer from sequelae. In all categories, an increased HR was found for patients with sequelae of COVID-19. This pattern was observed in both adults and older adults. Adult patients with sequelae of COVID-19 had an increased risk of thromboembolic events, with an adjusted HR of 14.50 (95% CI: 7.54–27.86), an increased risk for lung disease, with an adjusted HR of 33.81 (95% CI: 13.30–85.96) and infections, with an adjusted HR of 8.40 (95% CI: 4.49–15.70). Older adults patients with a diagnosis of sequelae of COVID-19 had an increased risk of thromboembolic events, with an adjusted HR of 12.50 (95% CI: 6.95–22.49), an increased risk for lung disease, with an adjusted HR of 9.83 (95% CI: 6.09–15.87), and an increased risk of infections, with an adjusted HR of 15.44 (95% CI: 10.47–22.78) (See Table 3).

## 4. Discussion

In this population-based Danish cohort study, which included all patients with a positive PCR test for COVID-19 between 1 March 2020 and 28 February 2022, we found a prevalence of 0.55% for sequelae of COVID-19 in both adults and older adults. The risk of being diagnosed with sequelae of COVID-19 increased with higher Charlson Comorbidity Index (CCI) scores in both age groups. While previous studies have demonstrated that increased comorbidity increases the risk of mortality in both the short and long term following COVID-19 infection [22,23,24,26,36]. Our study extends these findings with a two-year follow-up.

When subdividing the CCI into underlying comorbidities, we found an increased risk for sequelae of COVID-19 in both age groups for patients with either chronic pulmonary disease or rheumatologic disease. Conversely, dementia had a protective effect in our older adult age group for sequelae of COVID-19. Additionally, patients in both age groups with the diagnosis of sequelae of COVID-19 had an increased risk of being diagnosed with thromboembolic events, chronic lung disease, and infections, compared to patients without a diagnosis of sequelae of COVID-19.

The prevalence of sequelae of COVID-19 in our study—0.55%—is notably lower than estimates reported in other studies, which range from 6% to 55% [8,37,38]. This discrepancy is likely multifactorial. By including all patients with a positive PCR test, our study minimizes the risk of selection bias that may be present in other studies, though our study has another limitation in the diagnosis of sequelae of COVID-19. In Denmark, a national coordinated program centralized COVID-19 treatment to a few outpatient clinics. Only the most severe cases of sequelae were referred from general practitioners (GPs) to these specialized clinics [33]. Since only these clinics applied the ICD code for sequelae of COVID-19, our study does not include patients with milder cases, who were managed by their GPs. Consequently, our findings reflect only the most severe cases of sequelae of COVID-19, and the results should be interpreted accordingly.

The correlation between chronic pulmonary disease and sequelae of COVID-19 is well established. COVID-19 infection can lead to chronic lung lesions, such as pulmonary fibrosis in both severe and milder COVID-19 infections [15,39]. Our data support this link between chronic lung disease and sequelae of COVID-19, consistent with other studies where chronic obstructive pulmonary disease and asthma have been identified as risk factors for sequelae of COVID-19 [40].

Regarding rheumatic diseases and sequelae of COVID-19, it is evident that there is a correlation. Studies have found an increased risk of developing rheumatoid disease following COVID-19 infection [41,42] and an increased disease burden in patients with rheumatoid disease suffering from the sequelae of COVID-19 [43]. A dysfunctional immune system as well as increased T-cell activation up to two years after infection, has been theorized as the explanation [18,44]. Our data suggest that the correlation goes both ways, and that comorbidity with rheumatic disease increases the risk of developing sequelae of COVID-19, as has been found in another study [45].

Patients with a diagnosis of dementia have been shown to have an increased risk of hospitalization with COVID-19 [46,47] and an increased risk of mortality following COVID-19 infection [24,48]. Surprisingly, our study found dementia had a protective effect against sequelae of COVID-19. This finding may have multiple explanations. One meta-analysis documented that 10 out of 11 studies included reported patients with dementia were less likely to have access to medical services and received less optimal quality of care compared to patients not suffering from dementia [49]. In the same vein, symptoms of sequelae of COVID-19 include cognitive impairment [11], which may be difficult to distinguish from the normal pathophysiological progression of dementia. Both explanations could potentially lead to an underdiagnosis of sequelae amongst patients suffering from dementia.

Surprisingly, we found no correlation between congestive heart failure, renal disease, or diabetes and a diagnosis of sequelae of COVID-19. While all of these conditions have been correlated with higher hospitalization and mortality rates in acute COVID-19 infection [22], they did not correlate with an increased risk for sequelae of COVID-19 in either of our two age groups.

Studies on long-term cardiovascular outcomes after COVID-19 have shown an increased risk for thromboembolic disorders, sepsis, and pulmonary fibrosis following an initial COVID-19 diagnosis [17,50]. Our study further expands on this, showing that patients diagnosed with sequelae of COVID-19 have an increased risk of thromboembolic events, lung comorbidity, infections or sepsis following the diagnosis of sequelae of COVID-19 compared to other COVID-19 patients. While lung symptoms in patients with sequelae of COVID-19 have in part been attributed to direct lung damage [39,44], one study found that patients with sequelae of COVID-19 exhibited abnormal monocyte activation and migration in the lungs 3–9 months post-acute infection [51]. This increased activation correlated with both the severity of sequelae symptoms and radiological evidence of lung tissue injury. Similarly, although the increased risk of thromboembolic events can be partly explained by persistent coagulopathy following COVID-19 infection [21,52], increasing evidence suggests that a dysregulated immune system might play an equally important role. One study found a biochemical link between increased levels of proinflammatory cytokines and increased levels of circulating endothelial cells, suggesting that a dysregulated immune system leads to endothelial injury and increased risk of thromboembolic events [53]. Another study found that persistent activation of the complement system in patients with sequelae of COVID-19 leads to increased tissue injury and thereby increased thromboinflammation and endothelial dysregulation [21].

A theory explaining this dysregulation of the immune system is the virus reservoir theory, which posits that viral components may persist in the body long after the initial infection. One review article found persistence of COVID-19 RNA and proteins in the gut, lung tissue, and lymph nodes for months and, in some cases, even up to 1 year after infection. This prolonged viral exposure, a month after the initial infection, leads to increased production of proinflammatory markers as well as dysregulated adaptive immune system by altered differentiation of virus-specific T and B cells [54]. Ultimately, this sustained immune dysregulation could account for the observed increases in chronic lung disease, thromboembolic events, and infection risk among patients with sequelae of COVID-19.

This study has several strengths. The Danish population is relatively homogeneous, and the study, being a nationwide study based on well-established databases, eliminates reporting bias as well as geographical bias. Furthermore, the ability to secure complete follow-up data on all patients through these databases diminishes the risk of selection bias [55]. However, this study is not without limitations. In the registers applied in the current study, we did not have access to the socioeconomic and educational status of the patients and thus have not been able to adjust for this in our model. A recent study on the relationship between socioeconomic factors and the risk of being diagnosed with sequelae of COVID-19 found that ethnic minorities in Denmark had an increased risk of being diagnosed with sequelae of COVID-19. It furthermore documented that native Danes with a low level of education had a decreased risk of being diagnosed with sequelae of COVID-19 [56]. Thus, the lack of socioeconomic data in our study may confound our results.

Regarding the high HRs, residual confounding cannot be excluded, but we believe that the high HRs are most probably due to information bias. Furthermore, there is also a risk of surveillance bias, due to closer follow-up of patients with sequelae of COVID-19 in outpatient clinics and thereby increasing the HR in part B of our analysis. As such, the HR should be interpreted with caution, given the severity of the population receiving the B94.8A diagnosis.

Caution is warranted when extrapolating these findings to other populations. Denmark had a high vaccination compliance, in our study up to 85% were fully vaccinated prior to their infection. As a recent meta-study showed a decreased risk of sequelae of COVID-19 after vaccination [57] this could further explain the low prevalence of sequelae when compared to other international data, where vaccination had not been implemented as readily.

## 5. Conclusions

In conclusion, we document a prevalence of sequelae of COVID-19 of 0.55% in both adults and older adults. For both patients aged 40–59 years and 60 years and above, the risk of being diagnosed with sequelae of COVID-19 increased with increasing CCI. Furthermore, patients in both age groups had severely increased risk of developing either a thromboembolic event, lung disease, or infection following the diagnosis. This suggests that, in a clinical setting, these patients should be thoroughly examined if symptoms of these conditions arise.

## Figures and Tables

**Table 1:** Characteristics of Danish PCR-positive COVID-19 (first) according to adults (40–59 years) and older adults (≥60 years) patients from 1 March 2020 to 28 February 2022.

		Adults (40–59 Yrs)	Older Adults (≥60 Yrs)
Characteristic		n (%)	n (%)
N, Total		702,075	332,018
Age, median (25–75 percentiles), yrs		49 (44–54)	68 (63–75)
Time of year for PCR test			
	1 March 2020–31 August 2020	5626 (0.8)	4083 (1.2)
	1 September 2020–28 February 2021	55,513 (7.9)	30,547 (9.2)
	1 March 2021–31 August 2021	28,942 (4.1)	8481 (2.6)
	1 September 2021–28 February 2022	611,994 (87.2)	288,907 (87.0)
Sex			
	Male	330,357 (47.1)	160,161 (48.2)
Charlson Comorbidity Index score ^a^			
	No comorbidity (CCI = 0)	607,847 (86.6)	202,782 (61.1)
	Intermediate (CCI = 1–2)	83,642 (11.9)	95,541 (28.8)
	High (CCI ≥ 3)	10,586 (1.5)	33,695 (10.1)
Charlson Comorbidity Index (CCI) category ^b^			
	Congestive heart failure	2957 (0.4)	11,079 (3.3)
	Dementia	229 (0.0)	8728 (2.6)
	Chronic pulmonary disease	23,117 (3.3)	24,659 (7.4)
	Connective tissue disease	10,651 (1.5)	12,202 (3.7)
	Mild liver disease	5042 (0.7)	3260 (1.0)
	Hemiplegia	804 (0.1)	684 (0.2)
	Moderate-to-severe renal disease	4934 (0.7)	9546 (2.9)
	Diabetes with end-organ damage, Type 1 + 2	5971 (0.9)	9535 (2.9)
	Moderate-to-severe liver disease	560 (0.1)	745 (0.2)
COVID-19 vaccination before PCR-positive test			
	0	126,656 (18.0)	46,117 (13.9)
	1	9027 (1.3)	3782 (1.1)
	2	566,392 (80.7)	282,119 (85.0)

^a^ Charlson Comorbidity Index (CCI) calculated using 10 years of health data prior to the first positive PCR test. ^b^ A patient may be represented in more than one category in The Charlson Comorbidity Index.

**Table 2:** Sequelae of COVID-19 diagnosis according to CCI score in Danish PCR-positive COVID-19 (first) patients by adults (40–59 years) and older adults (≥60 years).

				Hazard Ratio	
Group/Outcome		Events n (%)	Time at Risk in Years	Crude HR (95% CI)	Adjusted ^a^ HR (95% CI)
Adults (40–59 y): Sequela, 2 years					
	CCI = 0 ^b^	3090 (0.5)	1,207,571.7	1	1
	CCI = 1–2	690 (0.8)	165,300.1	1.63 (1.50–1.77)	1.51 (1.39–1.64)
	CCI ≥ 3	107 (1.0)	20,306.7	2.04 (1.68–2.48)	1.85 (1.52–2.24)
Older (≥60 y): Sequela, 2 years					
	CCI = 0	902 (0.4)	398,619.5	1	1
	CCI = 1–2	627 (0.7)	178,698.7	1.53 (1.38–1.69)	1.54 (1.39–1.71)
	CCI ≥ 3	282 (0.8)	55,992.5	2.11 (1.85–2.41)	2.02 (1.76–2.32)

^a^ The models are adjusted for sex, age, number of vaccinations, time of PCR and hospitalization for COVID-19 infection (time-varying). ^b^ CCI: Charlson Comorbidity Index Score.

**Table 3:** Outcomes according to CCI score in Danish patients with sequelae of COVID-19 by adults (40–59 years) and older adults (≥60 years). The crude and adjusted hazard ratio of developing a thromboembolic, chronic respiratory or infectious disease in patients with COVID-19 sequelae compared to patients without sequelae of COVID-19 in a 2-year follow-up period.

				Hazard Ratio	
Group/Outcome		Events n (%)	Time at Risk in Years	Crude HR (95% CI)	Adjusted ^a^ HR (95% CI)
Risk of thromboembolic event, 2 years					
Adults (40–59 y):					
	Sequelae negative	75 (0.0)	1,393,124.0	1	1
	Sequelae positive	28 (0.7)	5166.0	462.00 (271.20–787.04)	14.50 (7.54–27.86)
Older (≥60 y):					
	Sequelae negative	83 (0.0)	633,282.2	1	1
	Sequelae positive	26 (1.5)	2407.2	341.59 (206.87–564.03)	12.50 (6.95–22.49)
Risk of being diagnosed with chronic lung disease, 2 years					
Adults (40–59 y):					
	Sequelae negative	29 (0.0)	1,393,141.1	1	1
	Sequelae positive	28 (0.7)	5241.1	676.51 (359.74–1272.20)	33.81 (13.30–85.96)
Older (≥60 y):					
	Sequelae negative	118 (0.0)	633,272.8	1	1
	Sequelae positive	100 (5.9)	2309.1	619.12 (450.77–850.35)	9.83 (6.09–15.87)
Risk of being diagnosed with infectious disease, 2 years					
Adults (40–59 y):					
	Sequelae negative	91 (0.0)	1,393,113.3	1	1
	Sequelae positive	28 (0.7)	5152.6	382.20 (224.89–649.53)	8.40 (4.49–15.70)
Older (≥60 y):					
	Sequelae negative	175 (0.1)	633,260.2	1	1
	Sequelae positive	122 (7.4)	2216.3	671.94 (509.05–886.96)	15.44 (10.47–22.78)

^a^ The models are adjusted for sex, age, number of vaccinations, hospitalization (time-varying), a diagnosis of outcome prior to COVID-19, and the Charlson Comorbidity score (CCI). In the analysis of chronic lung disease, any chronic pulmonary disease was omitted from CCI.

## Data Availability

Data used in this study are accessible in raw format by request from the Danish Health Data Authority (kontakt@sundhedsdata.dk). Access requires submission of an application for an individual research license. The authors of this study were not granted special privileges for acquiring the data.

## Supplementary Materials

The following supporting information can be downloaded at: https://www.mdpi.com/article/10.3390/jcm14227950/s1, Table S1: Risk of being diagnosed with sequelae of COVID-19 according to CCI category in Danish patients with a positive covid test.

## Abbreviations

The following abbreviations are used in this manuscript:

CCI	Charlson Comorbidity Index
HR	Hazard ratio
DNPR	The Danish National Patient Register
ICD	International Classification of Diseases

## Appendix A

The following ICD codes were used for outcome assessment.

Thromboembolic complication: I26.0 Pulmonary embolism with mention of acute cor pulmonale, I26.9 Pulmonary embolism without mention of acute cor pulmonale, I82.9 Embolism and thrombosis of unspecified vein, I80.3A Embolism in lower extremity, I82 Other venous embolism and thrombosis, I82.8 Embolism and thrombosis of other specified veins.

Respiratory disease: J43 Emphysema, J43.9 Emphysema unspecified, J44 Other chronic obstructive pulmonary disease, J44.8 Other specified chronic obstructive pulmonary disease, J44.9 Chronic obstructive pulmonary disease unspecified, J81 Pulmonary oedema, J84.1X Lung fibrosis, J84.1C Idiopathic lung fibrosis.

Infectious disease: J12 Viral pneumonia, not elsewhere classified, J15 Bacterial pneumonia, not elsewhere classified, J18.1 Lobar pneumonia, unspecified, J18.9 Pneumonia, unspecified, J13.9 Pneumonia due to Streptococcus pneumoniae J85.1 Abscess of lung with pneumonia J149 Pneumonia due to Haemophilus influenzae A41 Other sepsis.
