# Supplementary material for: Risk of Sequelae Following COVID-19 Infection: A Nationwide Study Focusing on Risk Factors and Long-Term Impacts
Source: J Clin Med. 2025 Nov 10;14(22):7950. doi: 10.3390/jcm14227950 (PMC12653031; doi:10.3390/jcm14227950)
Supplement: Supplementary file 1 [file jcm-14-07950-s001.zip › jcm-3945862-supplementary.pdf]

Table Supplement S1 – Risk of being diagnosed with sequelae of COVID-19 according to CCI category in Danish patients with a positive covid test.

	Exposed		Unexposed		Hazard ratios	
Group / Charlson Comorbidity Index category	Events n (%)	Time at risk in years	Events n (%)	Time at risk in years	Crude HR (95% CI)	Adjusted HR (95% CI) [a]
Adults (40-59y): Sequela, 2 years						
Congestive heart failure	21 (0.7)	5,753.9	3,866 (0.6)	1,387,424.6	1.30 (0.85-2.00)	0.73 (0.47-1.12)
Dementia[c]	-	-	-	-	-	-
Chronic pulmonary disease	262 (1.1)	45,526.2	3,625 (0.5)	1,347,652.3	2.14 (1.88-2.42)	1.35 (1.19-1.53)
Rheumatologic disease	97 (0.9)	21,067.8	3,790 (0.5)	1,372,110.7	1.67 (1.36-2.04)	1.25 (1.02-1.53)
Mild liver disease	49 (1.0)	9,832.2	3,838 (0.6)	1,383,346.3	1.79 (1.35-2.37)	1.00 (0.75-1.34)
Hemiplegia or paraplegia[c]	-	-	-	-	-	-
Renal disease	47 (1.0)	9,584.8	3,840 (0.6)	1,383,593.7	1.76 (1.32-2.34)	0.95 (0.71-1.28)
Diabetes with chronic complications	64 (1.1)	11,670.1	3,823 (0.5)	1,381,508.4	1.97 (1.54-2.53)	1.18 (0.91-1.52)
Moderate or severe liver disease	5 (0.9)	1,031.5	3,882 (0.6)	1,392,147.1	1.71 (0.71-4.11)	0.81 (0.33-1.99)
Older (≥60y): Sequela, 2 years						
Congestive heart failure	101 (0.9)	18,134.8	1,710 (0.5)	615,175.9	1.91 (1.56-2.34)	0.94 (0.76-1.16)
Dementia	17 (0.2)	12,365.3	1,794 (0.6)	620,945.4	0.43 (0.26-0.69)	0.36 (0.22-0.58)
Chronic pulmonary disease	264 (1.1)	43,673.6	1,547 (0.5)	589,637.1	2.25 (1.97-2.56)	1.26 (1.10-1.44)
Rheumatologic disease	112 (0.9)	22,465.5	1,699 (0.5)	610,845.2	1.77 (1.46-2.15)	1.40 (1.16-1.70)
Mild liver disease	35 (1.1)	5,843.0	1,776 (0.5)	627,467.7	2.07 (1.48-2.90)	1.30 (0.91-1.85)
Hemiplegia or paraplegia[c]	-	-	-	-	-	-
Renal disease	90 (0.9)	15,555.5	1,721 (0.5)	617,755.2	1.99 (1.61-2.45)	0.94 (0.75-1.17)
Diabetes with chronic complications	111 (1.2)	16,477.3	1,700 (0.5)	616,833.4	2.38 (1.96-2.88)	0.95 (0.78-1.16)
Moderate or severe liver disease	7 (0.9)	1,194.3	1,804 (0.5)	632,116.4	1.94 (0.92-4.07)	0.80 (0.37-1.75)

[a] CCI: Charlson Comorbidity Index.

[b] The models are adjusted for sex, age, number of vaccinations, hospitalization due to COVID-19 (time-varying), and for concomitant comorbid disease category from the CCI.
[c] Insufficient data for analysis.
